# Supplementary material for: Are carbon emissions trading and green financial instruments synergistic? -Comprehensive quantitative research based on content analysis
Source: PLoS One. 2024 Mar 7;19(3):e0298601. doi: 10.1371/journal.pone.0298601 (PMC10919601; doi:10.1371/journal.pone.0298601)
Supplement: S1 Data — (ZIP) [file pone.0298601.s001.zip › Data/地级市Z_F工作报告.zip!/─┌├╔╣┼/│α╖σ╩╨/└┤╫╘╣1⁄2╓┌║┼í╛┬φ┐╦╩2╛▌═°í┐_2018.docx]

各位代表：

　　现在，我代表市人民政府，向大会报告工作，请予审议，并请市政协委员和其他列席会议的同志提出意见。

　　一、过去五年工作回顾

　　市六届人大一次会议以来，面对复杂的宏观经济形势和繁重的改革发展任务，我们在以习近平同志为核心的党中央坚强领导下，全面贯彻落实上级党委、政府和市委决策部署，主动适应经济发展新常态，自觉践行新发展理念，与时俱进推动全面小康社会建设，团结拼搏，攻坚克难，奋力开创了全市经济社会持续健康发展的新局面。

　　综合经济实力持续增强。五年间，地区生产总值年均增长7.6%，全社会固定资产投资年均增长13.9%，社会消费品零售总额年均增长10%，城乡常住居民人均可支配收入年均分别增长9.3%和9.8%，这些重要指标年均增速均高于全区平均水平。在国家调整税收体制、强化质量管理的背景下，全市一般公共预算收入跃居全区第四位。千方百计增投资、稳基础、强产业，综合施策，主动作为，促进经济稳中有进、稳中提质。五年累计实施投资5000万元以上重点项目2260个，带动全社会固定资产投资完成6351亿元，比上个五年增加3430亿元；累计完成财政支出2007亿元，是上个五年的1.7倍；五年新增市场主体6万家，规上工业企业达到527家，新增农牧业产业化龙头企业130家。

　　转型升级步伐持续加快。坚持扩总量与调结构并举，多元发展、多极支撑的产业格局加快形成。工业集中做好新型化、集约化的加法和淘汰落后产能、减排降耗的减法。有色金属产业重点启动了两个40万吨铜冶炼项目入园扩建工程，新能源产业新增风电、光电装机超过200万千瓦，现代化工产业投产了一组煤化工、生物化工、硫酸化工等大项目，新兴产业加大了生物医药、电子信息、云计算等项目的引进培育；工业园区的集聚度不断增强，产值占全部工业比重达到47.6%；淘汰钢铁落后产能31.3万吨、煤炭落后产能615万吨，万元GDP能耗、水耗分别下降18.9%和13.6%。农牧业主攻调优结构和推动规模化、组织化、产业化经营。种植业和养殖业结构进一步优化，粮经饲比例由82:14:4优化为71:17:12，肉牛生猪存栏分别增加103.6万头和274.4万口；新增高效节水灌溉408万亩、高效节水经济林100万亩、设施农业71.7万亩；土地流转率、农牧民合作组织覆盖率、农畜产品精深加工转化率分别达到46.4%、50%和49.7%。服务业突出提档升级、创新突破。一批商业综合体投入运营，物流园区销售收入突破550亿元，电商进村实现全覆盖，9个旗县进入国家电子商务示范县行列；旅游业累计接待游客6400万人次，累计收入突破1000亿元；金融业营业规模大幅增长，新增各类机构25家，存贷款余额分别达到2100亿元和1600亿元，证券业成交额、保险行业保费收入累计实现3720亿元和253亿元，新增资本市场挂牌企业106家、实现直接融资122.6亿元。

　　发展保障能力持续提升。中心城市和旗县城关镇保持了发展强势。赤峰新区、桥北新区、松北新城建设突飞猛进，中心城区累计完成市政公用基础设施投资376亿元，建成区面积达到106平方公里，人口突破百万。9个旗县政府所在地建成区面积达到146平方公里。全市常住人口城镇化率由43.8%提高到49%。美丽乡村建设实现跨越式发展。累计投入资金211.2亿元，集中改造基础设施，全面提升公共服务水平，农村牧区生产生活条件得到极大改善，90%以上的嘎查村从中受益。重大基础设施工程多点发力、高效推进。新建改建各级公路9374公里，3条高速公路、10条一级公路建成通车，实现了县县通高等级公路、乡乡通等级公路、村村通油路，全市公路总里程达到2.7万公里；赤峰至京沈高铁全线开工，玉龙机场进出港旅客突破百万人次；农村牧区饮水安全、重点水库除险加固工程全面完成；新建66千伏及以上变电站45座、输电线路1460公里；启动了天燃气长输管道建设和宽带进乡村工程。生态保护与建设持续加强。制定了《赤峰市禁牧休牧和草畜平衡条例》，新增营造林面积739万亩，完成重点区域绿化128万亩，全市森林覆盖率达到35.6%，荣获国家森林城市、国家园林城市的称号。

　　改革开放持续深化。重点领域改革蹄疾步稳。放管服改革成效明显，市本级取消下放行政审批事项301项，比例达74.3%；商事制度改革走在全区前列，全面实施企业登记注册多证合一、一照一码、个体工商户两证整合和全程电子化登记改革；投融资体制改革初见成效，我市被国务院表彰为推广政府和社会资本合作模式成效明显、社会资本参与度较高的城市；农村牧区、国有企业、国有林场、供销合作、城市执法、教育考试招生制度、校园足球、文化体制、机构编制等专项改革有序推进；实施改革举措656项，形成改革成果309个。对外开放水平不断提升。五年累计引进资金超过2600亿元，保税物流中心封关运营，赤满欧国际班列开通运行；与朝阳、承德、锦州等毗邻地区战略合作深入开展；京蒙对口帮扶累计实施合作项目153个，到位资金5.4亿元。

　　民生社会事业持续改善。全市民生支出累计1542亿元，占财政支出的比重达77%。脱贫攻坚强力推进，累计投入各类扶贫资金300.5亿元，44万贫困人口实现稳定脱贫。就业和社会保障体系日益完善，累计新增城镇就业15.4万人,农村牧区劳动力转移就业352万人次；完成城乡医保整合，实现机关事业单位工作人员和城镇企业职工养老保险制度并轨。安居工程成效显著，建设保障性住房4863套，改造城市棚户区8.7万套、老旧小区640.3万平方米、农村牧区危房18.1万户，李克强总理关注的铁南棚户区改造全面完成、7300户居民迁入新居。社会事业加快发展，赤峰农博会晋升为国家级展会，新增自治区级以上科技创新载体129个；基础教育、职业教育、高等教育发展水平进一步提升，高考本科上线率始终保持全区领先；公立医院改革全面推开，药品加成全部取消，被评为全国基层中医药工作先进市；中心城区和旗县所在地新增文体场馆45.3万平方米，苏木乡镇文化站、嘎查村文化室实现全覆盖，红山文化遗址进入世界文化遗产预备名单，文艺创作、文明创建日趋活跃，全区第十三届运动会在我市成功举办。民族事务管理不断加强，民族教育水平进一步提升，蒙古语言文字推广工作位居全区前列。平安赤峰建设有效推进，安全生产、食药安全、生态环保、信访维稳、社会治安等工作深入开展，人民群众安全感和满意度进一步提升，荣获全国法治宣传教育先进市称号。社会科学、妇女儿童、残疾人、红十字、慈善等事业稳步发展，宗教事务、外事侨务、史志档案、防震减灾、气象服务、人防消防等工作不断加强。继续保持国家卫生城、全国双拥模范城等荣誉。

　　政府自身建设持续加强。四个意识不断强化，深入学习贯彻习近平治国理政新理念新思想新战略，坚定自觉地与以习近平同志为核心的党中央保持高度一致。法治政府建设扎实推进，制定并公布政府权责清单，全面推行政府法律顾问制度，强化行政监察和审计监督，坚持三重一大事项集体研究、会议决定，自觉接受人大、政协和社会各界监督，累计办理人大代表建议和政协委员提案1488件。创新型政府建设不断加强，建立了全市统一的公共资源交易平台，全面实施预决算公开、财政资金预评审制度，大力推行互联网＋政务服务，基本建成了市县乡村四级政务服务网络体系。廉洁政府建设日益深化，完成了公务用车制度改革，强化财政资金监管，三公经费支出大幅压缩。深入开展党的群众路线教育实践活动、三严三实专题教育、两学一做学习教育，严格执行中央八项规定精神和自治区党委、市委配套规定，政府系统作风进一步好转。

　　刚刚过去的2017年，是第六届政府的收官之年。一年来，我们全力以赴抓发展、惠民生、促改革、保稳定，较好地完成了市六届人大五次会议确定的工作任务。按照国家调整的统计核算口径，预计全市地区生产总值增长5.5%左右，500万元以上固定资产投资增长3%左右，社会消费品零售总额增长8%左右，城乡常住居民人均可支配收入分别增长8.5%和8%左右，几项主要经济指标增速均居全区前列。一般公共预算收入完成100.7亿元，财政收入质量进一步提高。在实际工作中，我们着力加强项目建设，启动实施了项目建设和招商引资冬季攻坚行动，全年动开工投资5000万元以上重点项目789个，当年完成投资1143亿元。着力推动工业振兴，出台了深化飞地经济、支持医药产业发展等推动实体经济发展、影响全局和长远的政策，全面启动规划22平方公里的马林有色金属产业园区建设，2×35万千瓦自备电厂和云铜40万吨铜冶炼项目开工建设，推动赤峰高新区晋升国家高新区，工业经济效益整体回升向好，规模以上工业企业利润率达到6%。着力推进农牧业供给侧结构性改革，引导压减了玉米和肉羊规模，有效增加了设施农业、人工种草、高效丰产经济林和生猪、肉牛养殖，农牧民参与产业化经营比率进一步提高。着力加快旅游业跨越发展，确立并实施三步走战略，组建市旅发委，启动1000公里旅游专线公路建设，签订1000亿元的旅游招商投资协议和框架协议，推动阿斯哈图石林晋升国家5A级景区。着力促进金融创新发展，出台支持金融产业发展1+6政策措施，进一步做大做实政府投融资平台，实施企业上市挂牌四年行动计划，启动塞北金融小镇建设，金融服务实体经济能力进一步增强。着力推动基础设施建设补短板，大板至经棚高速等9条高等级公路加快建设，赤峰高铁基本完成线下路基和桥梁工程，朝阳至赤峰天然气管道、琥珀沟水库开工建设，玉龙机场改扩建、东台子水库、小城子至平泉一级路、抽水蓄能电站等一批大项目前期工作取得积极进展。着力打造区域性中心城市，总投资378亿元、总规模3.8万套的中心城区集中连片棚户区改造工程启动实施，2×30万千瓦热电联产投入运行，中环快速路、高铁西站综合枢纽、综合管廊、垃圾发电、中水回用、环城水系治理、两山两园景观提升、20万千瓦背压机组热源等一批重大市政基础设施工程扎实推进；进一步理顺了新区行政管理体制，在全市范围内全面推行城市综合执法。着力推进脱贫攻坚工程，整合投入资金135.5亿元，突出重点、精准施策，5.8万贫困人口实现净脱贫。着力发展民生社会事业，市级主导实施了投资200亿元的十大民生工程，农村牧区中小学办学条件提升工程基本完成，中心城区11所中小学幼儿园全部开工；公共交通提档升级工程顺利推进，308辆新能源公交车投入运营；中国契丹辽博物馆、市医院改扩建、市综合检验检测中心、村村通油路、村村通宽带等工程加快建设；有效应对了8·11龙卷风灾害，388户灾民入冬前全部搬入新居；成功举办了中华一家亲·巴林草原情那达慕大会、全区乌兰牧骑艺术节。着力维护公共安全，制定并实施了安全生产1+8制度，生产安全事故起数和死亡人数同比分别下降54.6%和65.2%，认真开展了环保督察问题整改，启动了国家食品安全示范城市创建工作，推动解决三个重点群体信访问题，严厉打击各类违法犯罪行为，有力维护了社会大局和谐稳定。特别是，今年我们围绕庆祝自治区成立70周年，圆满完成了中央代表团接待工作，以迎促建推动全市各项事业发展，极大激发了广大干部群众的干事创业热情。

　　各位代表，过去五年全市经济社会发展取得的成就来之不易。这些成绩的取得，是以习近平同志为核心的党中央坚强领导的结果，是自治区党委和市委总揽全局、正确决策的结果，是市人大、市政协履职监督、鼎力支持的结果，是社会各界积极参与、群策群力的结果，是全市各族人民守望相助、团结奋斗的结果。在此，我代表市人民政府，向全市广大干部群众，向人大代表、政协委员和离退休老同志，向所有关心和支持赤峰发展的社会各界人士，致以崇高的敬意和衷心的感谢！

　　二、今后五年的发展愿景

　　新一届政府任期的五年，正值两个一百年奋斗目标的历史交汇期，与全国全区一道全面建成小康社会、乘势开启全面建设现代化赤峰新征程，是历史赋予我们的神圣使命，也是460万赤峰人民的殷切期盼，更是新一届政府必须完成好的政治任务。

　　当前，中国特色社会主义进入了新时代，我国社会的主要矛盾已经转化为人民日益增长的美好生活需要和不平衡不充分的发展之间的矛盾。站在新的历史方位，用新矛盾的重大判断来审视赤峰的发展，仍有很多困难和问题亟待解决，仍有很多矛盾和挑战不容回避。一是经济发展不平衡不充分，经济总量小，产业层次低，农牧业大而不强，工业初级化特征明显，服务业占GDP的比重偏低。二是民生保障不平衡不充分，人口基数大，农牧民和贫困人口多，有限的财政保障能力与较大的民生需求矛盾突出。三是城乡发展保障不平衡不充分，城镇化率偏低，生产要素布局分散，城乡公共服务欠账明显，基础设施建设短板多、融资难、压力大。四是生态环境改善不平衡不充分，总体遏制、局部好转的成果还不稳固，土地沙化、草原退化出现一定的反弹，河道径流减少、断流增多，地下水位下降的趋势明显加剧，水资源严重短缺的问题更加突出，生态保护与环境治理任重道远。五是社会治理改革不平衡不充分，12·3特别重大安全生产事故教训惨痛、殷鉴不远，企业生产、食品药品、社会治安、信访维稳等领域的公共安全仍然面临较大压力。面对发展不平衡不充分的市情和历史交汇期的艰巨任务，我们必须更加励精图治、拼搏进取，只有比别人流更多的汗、出更大的力，攻下硬堡垒、跑出加速度，才能保证小康路上不掉队，与全国全区一道同步跨上现代化建设新征程。

　　直面挑战，方能保持清醒；抢抓机遇，才能加速前行。党的十九大胜利召开，开启了中国特色社会主义事业新征程，置身新时代的大背景、大舞台，赤峰正在迎来新一轮加快发展的重要机遇期。世界经济同步复苏态势显著，中国经济持续稳中有进、稳中向好，成为拉动全球经济增长的主导力量；国家支持民族地区、贫困地区发展的政策持续加力，西部大开发、振兴东北、京津冀协同发展等国家战略和一带一路建设同步推进，我市将继续享受国家层面多重的政策利好；自治区出台了推动东部盟市跨越发展的实施意见，锡赤通经济区和蒙东能源基地建设上升为全区战略，我市将在统筹区域发展的政策中更多受益；随着高铁开通和通疆达海通道增多，我市的区位优势进一步彰显，接受京津冀城市圈和环渤海经济圈的辐射带动将更加直接，土地、原材料、劳动力等要素优势也将日益突出。更为重要的是，在党的十九大精神鼓舞下，全市干部群众谋发展、促发展、抓发展的愿望强烈，跨越崛起的决心和信心更加坚定。只要我们抓住机遇、奋发作为，就一定能够在这个充满希望的新时代里营造出新的气象，展现出新的作为。

　　今后五年政府工作的总体要求是：坚持以党的十九大精神为统领，坚持以习近平新时代中国特色社会主义思想为指导，统筹推进五位一体总体布局和协调推进四个全面战略布局，坚定不移地贯彻党中央、自治区党委和市委决策部署，坚定不移地贯彻稳中求进工作总基调和高质量发展根本要求，坚定不移地贯彻创新、协调、绿色、开放、共享的发展理念，着力抓重点、补短板、强弱项，着力实施七大战略，着力打好三大攻坚战，着力加快赤峰跨越崛起步伐，确保与全国全区一道全面建成小康社会，为开启全面建设现代化新征程奠定坚实基础。

　　今后五年工作的总体目标是：地区生产总值增速高于全区平均水平，城乡居民人均可支配收入增速高于经济增速；通过坚持不懈的努力，建成产业优势更加突出、城乡发展更加协调、发展活力更加旺盛、人民群众获得感更加实在、人与自然更加和谐的赤峰。

　　围绕上述总体要求和奋斗目标，今后五年市政府的工作将在以下五个方面聚焦用力：

　　（一）坚持传统产业升级与新兴产业培育同步推进，建设产业优势更加突出的赤峰

　　推动传统主导产业优化升级。围绕擦亮中国有色金属之乡品牌，加快矿业经济健康发展，推动国家重要的有色金属产业基地建设迈出新步伐；加快形成百万吨级铜冶炼优势产能，打造中国北方最大的铜冶炼深加工基地。围绕建设新型化工产业基地，发展壮大以煤、玉米、硫酸为原料的新型化工产业，形成更加完整的产业链条。围绕建设国家重要的绿色农畜产品生产加工输出基地，注重品种改良、品质提升、品牌塑造，突出城镇近郊田园综合体建设，加快农牧业与二三产业融合发展，集中打造一批绿色有机产品品牌。围绕建设具有区域竞争力的医药产业基地，全面落实各项扶持政策，引进和培育一批新药生产项目，尽快把医药产业打造成为支柱产业。围绕激发建材、纺织产业竞争力，推进现有企业引联重组，促进传统产业焕发新活力。

　　推动新兴产业重点破题。制定完善全市战略性新兴产业发展规划，出台重点新兴产业扶持政策，设立产业发展基金，下力气引进和培育新能源、新材料、节能环保、信息技术、生物科技等新兴产业。加快建设京津冀清洁能源保障输出基地，努力落实京津冀千万千瓦电力消纳协议，着力打通赤峰能源外送通道，切实增强新能源产业的发展后劲。积极发展数字经济，大力培育互联网+、大数据和人工智能产业，加快推进电子信息和动漫制作等产业，推动信息化与工业化融合发展。力争到2022年，全市战略性新兴产业增加值占GDP比重超过10%。

　　推动高端服务业创新突破。深入推进旅游三步走战略，集中打造以克什克腾旗为核心的草原风光观光游、以巴林左旗为核心的契丹辽文化体验游、以翁牛特旗为核心的大漠风光极限运动游、以宁城县和喀喇沁旗为核心的休闲度假游、以中心城区为核心的近郊乡村游、以敖汉旗为核心的史前文化研学游六张旅游王牌，构建完善的自驾游服务体系，积极推进特色小镇建设，建成中国草原自驾游胜地和中国旅游强市。大力发展现代物流业，深入推进传统商贸服务业迈向信息化、高端化，把中心城区打造成区域性现代商贸物流中心。深入推进金融业创新发展，引进新型金融机构，加快企业上市融资步伐，推动区域股权交易发展，健全金融服务体系，积极发展普惠金融，推动塞北金融小镇形成特色和规模。深入推进文化产业发展，扶持康养健身、休闲娱乐等生活性服务业向精细化和高品质转型升级。

　　推动园区经济集约发展。按照企业集中、产业集聚、经营集约的思路，举全市之力打造赤峰东部工业走廊，深入落实飞地经济政策，引导加工和制造业项目向东部工业经济走廊有序流入、合理布局，形成若干特色产业集群，挺起赤峰工业脊梁。推动各地园区向专业化、特色化发展，加大基础设施投入，不断提升园区的支撑力和竞争力。

　　（二）坚持新型城镇化与乡村振兴同步推进，建设城乡发展更加协调的赤峰

　　全力打造宜居宜业城镇。有效提升中心城区综合承载能力，统筹新老城区发展，全面完成中环快速路、河道治理、综合管廊、供热扩能、污水垃圾处理等一批重大市政基础设施工程，以管网更新、交通疏堵、空间增绿、风貌治乱、业态提升为切入点改造老城区，不断完善中心城区运行保障。有力推动国家生态园林城市建设，实施一批中心城区绿亮美工程和景观园林工程，到2022年建成区绿化覆盖率达到40%，不断提高生态宜居水平。有序推进重点小城镇特色发展，按照城市标准建设城关镇，打造一批承载能力强、特色鲜明的重点小城镇，构筑龙头突出、梯次清晰、带动有力的新型城镇发展格局。到2022年全市常住人口城镇化率达到55％以上。

　　全力实施乡村振兴战略。按照产业兴旺、生态宜居、乡风文明、治理有效、生活富裕的目标，制定乡村振兴工作规划，统筹做好三农三牧工作，加快农牧业和农村牧区现代化建设步伐。大力发展现代农牧业，落实好第二轮土地承包到期后再延长30年政策，深化农牧业供给侧结构性改革，积极推广资源变资产、资金变股金、农牧户变股东的三变模式，发展多种形式的适度规模经营，提高农牧民组织化程度，培育壮大新型经营主体，建立现代农牧业生产经营体系，不断提高农牧业质量效益。持续改善农村牧区生产生活环境，巩固美丽乡村建设成果，继续提升农村牧区基础设施和基本公共服务水平。加强农村牧区精神文明建设，积极倡导移风易俗，大力传播新思想、新文化、新科技，着力培育新型农牧民。推动农村牧区治理改革，加强农村牧区基层基础工作，锻造三农三牧工作队伍，规范村规民约，健全自治、法治、德治相结合的乡村治理体系。建立完善的政策保障机制，多渠道增加农牧民收入，逐步缩小城乡居民收入差距。

　　全力改善城乡发展基础条件。统筹城乡一体化发展，着力推进一批重大基础设施建设工程，形成适度超前、布局合理、配套完善、满足发展需求的基础设施网络体系。完善公路网，建设高等级公路1300公里以上，加快构建六横八纵骨干公路框架，进一步织密农村牧区公路网，建好、管好、护好、运营好农村牧区公路。完善铁路网，完成5条铁路续建改造工程，确保赤峰高铁2019年建成通车，开通赤大白铁路客运。完善航空网，完成玉龙机场改扩建工程和林西支线机场新建工程，建成低空飞行网络。完善信息网，加快智慧城市建设，全面完成网络光纤村村通工程，实现移动通信网络全覆盖、无盲点。完善能源网，新建220千伏及以上变电站5座，建成朝阳至赤峰天然气长输管道工程。

　　（三）坚持改革创新与对外开放同步推进，建设发展活力更加旺盛的赤峰

　　实施创新驱动战略。强化企业创新主体地位，加强政策引导，激活企业创新发展的内生动力，到2022年，新增高新技术企业100家以上。强化科技创新，围绕产业链部署创新链，努力在重点产业领域研发引进一批核心技术，集中力量推动高新区产业上台阶、上水平，建设蒙东区域创新示范区。强化大众创业、万众创新，持续打造创业孵化基地、小微企业园等创新发展平台。强化人才引进，推进玉龙人才工程，启动天南地北赤峰人返乡创业行动计划，全力打造人才高地。

　　深化重点领域改革。抓好供给侧结构性改革，优化存量资源配置，扩大优质增量供给，不断增强竞争优势和发展后劲。抓好以放管服为重点的商事制度改革，全面实行市场准入负面清单制度，营造公平竞争的营商环境，充分激发各类市场主体投资活力。抓好投融资体制改革，充分发挥国有资本运营公司功能，规范推广政府与社会资本合作模式，扩大社会有效投资。抓好农村牧区改革，完成土地草牧场确权登记，完善三权分置制度，健全土地草牧场流转服务体系，逐步壮大嘎查村集体经济，有效破解农村牧区产业发展的体制机制障碍。

　　全方位扩大对外开放。以大开放促进大发展，倾力做好融入京津、拓展东北、向北开放、转身向海四篇文章。深入对接京津冀协同发展战略，主动承接产业链整体转移和关联产业协同转移，着力打造自治区融入京津冀协同发展先行区。加强与东北经济区的对接联系，积极参与产业分工协作，推动基础设施互联互通、重点产业协调发展。主动融入国家一带一路建设，加快完善通疆达海的铁路公路通道，密切与满洲里、二连浩特等口岸城市的合作，大力推动赤峰保税物流中心晋升为国家综合保税区。深化与锦州港、天津港的战略合作，改进陆港联运、公铁联运，加快推动通关便利化，打造综合性、多功能物流运输平台，建设赤峰国际物流港，提升向沿海发达地区开放、向欧亚市场开放的能力。坚持不懈招商引资，瞄准发达地区、关联产业、知名企业、一流院所，围绕做大总量和提高质量大力引智引业，创建国家级承接产业转移合作示范区。

　　（四）坚持保障改善民生和创新社会治理同步推进，建设人民群众获得感更加实在的赤峰

　　坚决打赢精准脱贫攻坚战。深入落实精准扶贫、精准脱贫基本方略，既不降低标准，也不吊高胃口，整合各方面资源汇聚脱贫攻坚主战场，注重提升贫困群众内生动力和发展能力，为永久脱贫提供根本保障。加大财政投入力度，创新金融扶贫机制，动员社会力量参与，突出抓好产业扶贫、健康扶贫、易地搬迁扶贫，确保到2020年现行标准下农村牧区贫困人口稳定脱贫，贫困旗县区全部摘帽。

　　持续提高就业增收水平。坚持就业优先战略，实现更高质量和更充分就业。加强职业技能培训，重点抓好新成长劳动力培训，造就一批高素质产业大军。鼓励创业带动就业，大力发展市场化、专业化众创空间和双创示范基地。做好困难群体就业帮扶工作，促进高校毕业生、农民工等群体多渠道就业创业。通过千方百计增加就业，切实巩固城乡居民持续增收的基础。

　　发展公平而有质量的教育。落实立德树人根本任务，推进教育综合改革，推动教育实现内涵发展。着力增加学前教育供给，构建以公办园为主体、普惠制民办园为补充、适龄幼儿全覆盖的学前教育体系，力争到2022年普惠性幼儿园覆盖率达到90%。着力促进基础教育均衡，推动优质教育资源合理配备，构建城乡一体化发展新格局，实施高中带动战略，推动基础教育优质特色多样化发展。着力提升职业教育质量，深入推进自治区职业教育实验区建设，优化职普结构，深化产教融合、校企合作，提升就业能力。着力抓好民族教育覆盖，认真落实优先发展民族教育政策，重点办好蒙古族群众聚居区和蒙古族生源较多地区的民族教育，有效满足蒙古语授课需求。着力办出高等教育特色，完善市属高校重大决策形成机制，强化高校发展与全市大局的联系和呼应，推动赤峰学院向应用型大学转变，支持交通职业技术学院与工业职业技术学院打造特色专业品牌，更好地服务地区经济社会发展。

　　扎实推进健康赤峰建设。深化医药卫生制度改革，加快建立基本医疗卫生制度和优质高效的医疗卫生服务体系，提高优质医疗服务供给水平。加强计生服务管理，促进人口均衡发展。强化食品药品安全监管，推进信息化全过程管控，健全农畜产品质量可追溯体系，建成国家食品安全示范城市，保障人民群众舌尖上的安全。树立大卫生大健康观念，推进卫生与健康事业发展，广泛开展全民健身活动，努力提高全市人民健康水平。

　　加快建设文化强市。以创建全国文明城市为引领，深化群众性精神文明创建活动，驰而不息推动社会主义核心价值观进学校、进社区、进企业、进军营、进农村牧区、进机关。加快发展文化事业，不断完善城乡公共文化服务体系，深入实施文化惠民工程，丰富群众性文化活动。强化文艺院团基础能力建设，弘扬乌兰牧骑精神，扎根基层、服务群众，创作一批接地气、传得开、留得下的文艺作品，满足群众多样性文化需求。重视历史文化资源保护，深入实施红山文化、契丹辽文化和蒙元文化保护开发，力争完成红山文化遗址、辽代上京城和祖陵遗址申遗工作。大力发展文博文创产业，积极培育文化消费市场，加快建设特色园区和产业基地，力争到2022年文化产业增加值占GDP比重达到5%以上。

　　不断提升社会保障能力和水平。坚持既尽力而为，又量力而行的原则，合理引导社会预期，加快建设多层次、可持续的社会保障体系。完善基本养老保险制度，实施全民参保计划，配合做好养老保险全国统筹工作。加强社会救助体系和慈善公益体系建设，加大对特殊人群关爱保护力度，全力保障困难群体的基本生活。积极应对人口老龄化问题，加快发展老龄事业和养老服务业，积极构建养老、孝老、敬老政策体系和社会环境。加快建立多主体供应、多渠道保障、租购并举的住房制度，全面完成中心城区集中连片棚户区改造和农村牧区危房改造，多渠道改善各类人群的住房条件。

　　坚决打赢防范化解重大风险攻坚战。按照摸清底数、严控增量、分类施策、压实责任的要求，积极稳妥化解政府债务，确保到2021年综合债务率降到合理区间。严格规范政府投资项目、举债融资、基金运作和购买公共服务行为，从严控制政府债务增量。加强金融安全管控，坚决打击非法集资等违法违规金融活动，有效防范化解银行信贷风险，密切关注房地产、国有企业债务等方面的风险，加强农村牧区高利贷综合治理，确保不发生区域性金融风险。

　　全力营造安全稳定的发展环境。牢固树立大安全观，加强和创新社会治理，扎实推进平安赤峰、法治赤峰、和谐赤峰建设，打造共建共治共享的社会治理格局。健全完善安全生产风险防控和隐患治理常态化机制，从源头上预防和遏制重特大安全事故发生。认真落实党和国家民族政策，加强民族团结进步事业，依法管理民族宗教事务。强化国防动员和后备力量建设，深化双拥共建活动，推动军民融合发展。统筹抓好矛盾化解、信访维稳、社会治安、防灾减灾等工作，确保人民群众安居乐业、社会大局长治久安。

　　（五）坚持生态保护与环境治理同步推进，建设人与自然更加和谐的赤峰

　　践行绿色发展理念。大力发展绿色经济，实行最严格的生态环境保护制度，推动各行业各领域绿色化生产。严守产业准入的环保底线，绝不接受能耗排放不达标的产业项目，建立各类保护区工矿企业有序退出机制和绿色工矿企业达标制度。加强资源节约型和环境友好型社会建设，引导广大群众节约资源、保护环境，推动形成绿色发展的浓厚氛围。

　　坚决打赢污染防治攻坚战。实施大气污染防治行动，打赢蓝天保卫战。加强水污染防治，坚持系统治水，全面落实河长制，实行从水源到水龙头全过程监管，促进生活和生产污水达标排放。强化土壤污染管控和修复，加强农业面源和重金属污染防治，推进化肥、农药减量化使用，加强固体废弃物和垃圾处置。实施老哈河上游生态保护与环境整治三年行动计划，集中力量、重点突破，全力遏制区域生态环境状况恶化的势头，迈出拯救母亲河行动的关键一步。

　　加大生态修复和治理力度。坚持保护优先原则，严格划定生态保护红线、永久基本农田、城镇开发边界三条控制线，将开发强度和控制线落地上图，推动经济社会发展由外延扩张向内涵提升转变。严格执行《赤峰市禁牧休牧和草畜平衡条例》，依法保护森林草牧场，扩大耕地轮作试点范围，推动森林草原湿地生态系统自然修复。实施重大生态建设工程，完成林草种植、防沙治沙等六个百万亩基地建设。力争到2022年，全市森林覆盖率达到36％以上。

　　突出解决水资源严重短缺的问题。正视我市水资源保障与用水需求矛盾日益加剧的严峻现实，坚持开源与节流并举、以节流为主的方针，切实在提高水资源保障能力上取得实质性进展。加强重点水源工程建设，确保东台子水库、琥珀沟水库、跨区域调水、城市引供水等一批重大工程建成投运。突出抓好农业节水改造，严格控制新增灌溉面积，下力气推广节水作物种植和农业节水技术，探索建立农业用水定额管理机制。加快推进节水型城市建设，推广再生水利用和中水回用，全面完成中心城区自备水源井关井压采工作，深化阶梯水价制度改革，显著提升中心城区水资源节约利用水平。认真实行最严格的水资源管理制度，出台并严格执行《赤峰市地下水资源保护条例》，坚决遏制水资源过度开发和粗放利用。

　　三、2018年工作重点

　　2018年是新一届政府工作的起步之年，是深入贯彻落实党的十九大精神的开局之年，也是决胜全面建成小康社会的关键之年，做好今年工作意义十分重大。

　　综合考虑当前和长远、需要和可能、速度和质量等诸多因素，今年全市经济社会发展主要预期目标是：地区生产总值增长7%左右，一般公共预算收入力争增长6%左右，规模以上固定资产投资增长9%左右，社会消费品零售总额增长8.5%左右，城乡常住居民人均可支配收入分别增长8%左右和8%以上，居民消费价格涨幅3%左右；新增城镇就业2.8万人以上，城镇登记失业率控制在4%以内；全面完成节能减排目标任务。

　　围绕完成上述目标，我们将重点做好以下八个方面工作：

　　（一）全力推动三大攻坚战深度破题

　　坚决打好防范化解重大风险攻坚战。强化金融监管，规范金融市场管理，严厉打击非法集资、金融诈骗和逃废银行债务行为，开展好互联网金融专项整治，确保不发生区域性金融风险。严格规范政府债务管理，按照借之有度、用之有效、还之有道的方针，依法依规解决资金短缺问题，把政府债务分门别类纳入全口径预算管理，按计划完成年度债务化解任务，确保公共财政安全。加快社会征信体系建设，完善综合信用评价系统，建立严重失信黑名单和信息共享披露制度，健全失信制裁和社会联防机制，增加失信成本，弘扬诚信文化，从社会面减少各类信用风险。

　　坚决打好精准脱贫攻坚战。牢牢把握六个精准、五个一批总要求，落实市县乡村脱贫攻坚责任，整合投入各类扶贫资金150亿元，年内确保7.7万贫困人口稳定脱贫。进一步加强产业扶贫，充分利用三变机制，将更多的贫困户纳入龙头企业以及合作组织的覆盖范围，建成扶贫产业园25个，稳定主导产业对贫困人口的增收贡献。进一步加强易地搬迁扶贫，抓好配套产业建设，确保贫困人口搬得出、稳得住、能致富。进一步加强健康扶贫，实施三个一批行动计划，全面落实医保倾斜政策和商业补充医保制度，有效解决因病致贫、因病返贫问题。进一步加强教育扶贫，积极落实资助政策，减轻就学负担对贫困家庭的拖累。进一步加强金融扶贫，落实扶贫小额信贷、强龙贷等扶贫贷款40亿元。进一步加强扶贫资金监管，规范财政专项扶贫资金管理使用，确保扶贫资金精准惠及贫困人口。

　　坚决打好污染防治攻坚战。深入实施大气、水、土壤等污染防治行动计划，确保蓝天保卫战取得全面胜利，水资源和土壤污染防控取得积极进展，有序退出国家自然保护区内工矿企业。认真抓好全国第二次污染源普查工作。启动总投资106亿元的老哈河上游生态保护与环境整治三年行动计划，实施生态修复、农业节水、废污处理、矿山整治、城市节水、水生态保护6大工程，年内完成生态修复20万亩、农业节水改造40万亩、污水集中处理乡镇10个、退出整治流域内矿山企业4个、疏浚河道232公里。严格落实《赤峰市禁牧休牧和草畜平衡条例》，严肃治理过度放牧行为，用法治手段推动自然生态稳定好转。推进生态文明制度改革，落实草原、湿地、沙化土地保护和修复制度，配合完成《赤峰市地下水资源保护条例》立法工作。加大生态建设力度，完成营造林100万亩、京津风沙源治理38万亩、退耕还林18万亩、重点区域绿化10万亩、水土保持综合治理150万亩。

　　（二）全力推动项目建设再上台阶

　　坚持把项目建设作为推动赤峰跨越崛起的关键抓手，全市动员、全民参与，集中精力抓投资、上项目、稳增长，实施投资5000万元以上重点项目700个以上，完成投资1300亿元以上。深入开展招商引资冬季行动，围绕主导产业，突出重点地区，着力引进一批符合环保政策、带动产业升级、促进就业创业的大项目、好项目，确保引进投资亿元以上的产业项目100个以上。高度重视营商环境的改善提升，把抓项目与抓环境统筹起来，切实解决具体项目落地条件，认真落实支持非公经济发展各项政策措施，大力营造社会面亲商、安商、富商的浓厚氛围。积极争取国家和自治区支持，加大生态环保、社会事业和基础设施投入力度，力促一批事关全市长远发展的重大工程落地开工。落实鼓励支持扩大民间投资的政策措施，在基础设施和公共服务领域规范推广政府与社会资本合作。

　　（三）全力推动工业经济焕发活力

　　围绕集中打造赤峰东部工业经济走廊，深入落实飞地经济政策，构建一区四园集约发展平台，完成园区道路主网架、铁路专用线、污水处理厂、固废渣场和危废处理厂等基础设施建设，力促2×35万千瓦自备电厂建成投运，推动高新技术产业园区承载能力有效提升。围绕做大做强有色金属产业，集中精力推进工业项目建设，确保赤峰云铜40万吨铜冶炼项目竣工投产，加快金剑铜业搬迁扩建，集中引进和建设一批铜产业精深加工、硫酸化工等项目，促进产业增链、循环发展，推动有色金属产业快上台阶。围绕升级壮大医药产业，设立医药产业发展引导资金，落实扶持医药产业发展壮大的奖励办法，鼓励引导医药企业开发新产品，引进高端人才和国家新药生产线，加快普因医药、泰领海创生物科技园项目建设，推动医药产业增添后劲。围绕形成产业发展新支撑，开工建设70万吨乙二醇及120万吨铬铁合金、2×30万吨燃料乙醇、120万千瓦抽水蓄能、60万吨缓控释肥、正威电子信息产业园等一批重大工业项目，确保远联钢铁技改升级、拓佳液晶显示屏二期、凌云海制糖、安琪酵母等90个重点项目竣工投产，形成更多壮大工业规模的有生力量。围绕促进实体经济健康发展，创新贷款担保合作机制，发挥政府担保平台功能，引导金融机构助推实体经济发展，有效缓解中小微企业融资难问题，全年新增贷款160亿元；强化企业运行调度，切实加强服务保障，综合施策降低企业成本，通过引联重组、技改升级等方式推动有条件的停产企业、亏损企业走出困境，促进工业经济稳健增长。

　　（四）全力推动服务业实现重点突破

　　实施旅游六张王牌行动计划，全年动开工涉旅项目260个，完成涉旅投资100亿元，突出抓好美林谷国家级旅游度假区和玉龙沙湖5A级景区创建工作，完成1000公里旅游专线公路建设，确保旅游大厦等重点项目投入运营；加快旅游配套服务体系建设，密切与京津冀和周边地区的联动合作，加快构建汽车自助服务系统；集中攻坚实现旅游景区移动通信网络全覆盖，完成移动客户端游客服务中心建设；办好旅游推介招商、体育品牌赛事等重大活动，不断提升赤峰旅游的知名度和影响力。促进商贸物流产业转型升级，稳定提高对外贸易总额，推进赤峰商贸物流城一期、金融物流港二期建设，加快蒙东农机市场、利丰汽车市场、千禧建材市场、大福建材市场等退城入园步伐，推动保税物流园区晋升国家综合保税区，提升电子商务进农村示范县建设水平。积极扶持培育金融产业，推动华夏银行在我市设立分支机构，加强非标准有色金属交易中心招商工作，吸引更多金融企业入驻塞北金融小镇，深化企业上市挂牌四年行动计划，积极拓宽并购重组、融资租赁等融资渠道，力促实现直接融资50亿元以上。

　　（五）全力推动乡村振兴扎实起步

　　围绕促进产业兴旺，立足实际深度调整优化种养业结构，加快发展设施农业和高效节水农业，持续发展特色种养业和农畜产品加工业，下力气推动田园综合体建设取得突破性进展，促进农牧业与二三产业有机融合，全年新增设施农业、优质紫花苜蓿、丰产经济林各20万亩，新增节水灌溉面积40万亩，优质肉牛肉羊出栏900万头只以上、生猪出栏700万口以上。围绕促进生态宜居，继续完善农村牧区基础建设，探索农牧业废弃物处理、农村牧区垃圾处理、农牧户厕所改造新模式，出台农村牧区环境治理管护长效机制。围绕促进乡风文明，深入开展文明村镇、文明家庭、道德模范等评选活动，大力倡导和睦邻里、孝老敬亲的社会风气，加快推动婚丧嫁娶等领域的移风易俗。围绕促进治理有效，加强法治宣传教育，积极推行村务公开、财务公开，做好第十届嘎查村换届工作。围绕促进生活富裕，认真落实各项惠农惠牧政策，加大农牧民生产技能培训力度，引导富余劳动力转移就业。

　　（六）全力推动基础设施建设整体跃升

　　实施重大基础设施建设前期工作集中攻坚，推动一批重大基础设施工程顺利启动建设。公路建设投资64.1亿元，加快推进15个续建项目，确保10个项目年内建成通车，新开工建设小城子至平泉一级路和十家至黑里河二级路，力争赤峰至小卡拉等9个公路项目具备开工条件。铁路建设投资53.5亿元，加快赤峰高铁建设，确保年内进行轨道铺设，完成京通线电气化改造和叶赤铁路扩能改造，开工建设集通铁路电气化项目。民航建设投资5亿元，开工建设玉龙机场改扩建工程和5个通用机场，加快推进林西支线机场前期工作。中心城区市政基础设施建设投资100亿元，完成环城水系治理、三座店水库向中心城区引供水、综合管廊、两山两园景观提升等工程，加快推进中环快速路、高铁西站交通枢纽、中水回用配套管网等在建项目，开工建设水源地外迁、第四水厂、10万吨再生水厂、红庙子污水处理厂二期以及6座跨河桥梁等项目。选择重点领域实施老城区改造。全面加强城市管理，提高智慧化、精细化管理水平。能源通道建设投资110亿元，确保朝阳至赤峰天然气长输管道、城乡电网新建改造、富龙热电联产等项目竣工投运，推动赤峰至华北电力外送通道前期工作取得实质性进展。水利建设投资20亿元，加快琥珀沟水库建设，开工建设跨区域调水赤峰支线、东台子水库和47座病险水闸除险加固工程。

　　（七）全力推动民生社会事业更好发展

　　抓好创业赤峰工程，充分发挥安快创业谷以及各类双创平台作用，支持企业异地孵化和成果转化，做好复转军人、大学生、农牧民和就业困难人员就业再就业，确保年内城镇新增就业2.8万人以上，城镇零就业家庭保持动态清零。严格落实各项社会保障政策，继续提高城乡困难群众救助标准，全力保障困难群众基本生活。推进棚户区改造工程，投资110亿元，改造城市棚户区1.32万套、185万平方米。大力发展科技事业，办好中国北方农博会，实现赤峰高新区晋升为国家级高新区。统筹推进各类教育加快发展，完善蒙古语授课学前教育和基础教育办学体系，在赤峰新区新建一所蒙古语授课实验小学，继续实施全面改薄计划，启动实施高中普及攻坚计划，确保所有旗县通过国家义务教育均衡发展认定，加快赤峰学院全国产教融合试点校建设。加快发展卫生事业，推进城市公立医院综合改革，强化三医联动，全面推行药品采购两票制，加强重大疾病防控，加快市医院改造提升工程，迁址新建市传染病医院、市蒙中医院和中心血站，确保综合检验检测中心建成运营。深入实施文化惠民工程，充分发挥乌兰牧骑和乡村文艺小分队作用，积极开展文艺院团大学习、大宣传、大排练、大演出行动，强力推动文化活动下基层；加强文化遗产保护利用，启动辽上京、辽祖陵、辽庆陵、二道井子夏家店下层文化和辽中京大明塔五大国家考古遗址公园规划建设，完成中国契丹辽博物馆、红山文化展示中心、档案馆主体工程。加快推进厕所革命，分类制定城市、农村牧区、旅游景区厕所建设规划，明确建设、改造、运营机制，确保年内取得实质性进展。

　　（八）全力推动公共安全持续向好

　　严格落实安全生产系列政策和各项规定，强化底线思维，坚持警钟长鸣，建立安全生产大检查常态化机制，推进安全生产标准化和信息化建设，拓展安全生产社会化服务渠道，保持严管重罚高压态势，坚决防止重特大事故发生。强化食品药品监管，加快推进国家食品安全示范城市创建工作，全面完成旗县区食品小作坊集中加工园区和标准化农贸市场建设，完善食品安全检验检测体系，确保人民群众饮食用药安全。严格落实粮食安全市长责任制。及时就地解决群众合理诉求，妥善处理各类信访矛盾。深化平安赤峰建设，完善社会治安立体防控体系，开工建设雪亮工程，严密防范和严厉打击各类违法犯罪活动，确保社会大局和谐稳定。健全突发事件应急处置机制，提高气象预测、防灾减灾和应急救援能力。

　　四、新时代政府自身建设

　　对标新时代、新思想、新任务、新要求，政府的治理体系和治理能力还存在诸多不适应，形式主义、官僚主义还有一些新表现，思想解放不够、执行能力不强、服务意识淡薄的问题还比较突出，个别领域损害群众利益的现象还时有发生。面对全面从严治党的政治要求和攻坚克难的发展重担，新一届政府必须更加自觉地维护以习近平同志为核心的党中央权威和集中统一领导，更加自觉地树牢四个意识，更加自觉地坚守政治纪律和政治规矩，更加自觉地改进作风、加强自身建设，以立党为公、执政为民的新形象，在推动全市经济社会持续健康发展的实践中展现更大作为、交出优异答卷。

　　（一）强化宗旨意识，建设服务型政府。牢固树立以人民为中心的理念，把广大人民群众是否满意作为衡量政府工作得失的根本标准。进一步转变政府职能，深化简政放权，创新监督方式，增强政府执行力和公信力。持续深化放管服改革，加强政务公开，扩大公众参与，拓展社会监督，优化政府服务，为促进就业降门槛，为市场主体减负担，为激发投资拓空间，为公平营商创条件，为群众办事增便利。

　　（二）强化法治思维，建设法治政府。牢固树立宪法法律至上观念，坚持法无授权不可为、法定职责必须为的原则，严格按照法定程序和内容履行职责、推动工作，严格实行科学民主决策，认真执行政府重大问题集体决策、社会公示和听证制度。自觉接受人大的法律监督和工作监督，认真执行人大及其常委会的决定，全面接受政协的民主监督，加强协商民主，保持与民主党派、工商联的经常性联系，广泛听取人民群众意见建议，切实提高决策的民主化、科学化水平。全面加快依法治市进程，深入开展七五普法，增强运用法治思维和法治方式深化改革、推动发展、化解矛盾的能力。

　　（三）强化担当精神，建设效率政府。坚持发展第一要务，以经济建设为中心，勇于打破常规，敢于求变突破，不因包袱重而等待、困难多而不为，不因有风险而躲避、有阵痛而不前，扑下身子抓落实、撸起袖子加油干，稳扎稳打、步步为营，脚踏实地把各项工作推向前进。进一步提高工作效率，让广大干部从文山会海中解脱出来，拿出更多精力抓落实。强化绩效考核和效能监督，坚决纠正不作为、慢作为、乱作为等现象，健全激励机制和容错纠错机制，保护和激发广大干部想干事、敢干事、干成事的热情，以时不我待、只争朝夕、勇往直前的精气神，奋力开创新时代赤峰跨越崛起的新境界。

　　（四）强化作风改进，建设廉洁政府。坚持一岗双责，强化红线意识和底线思维，认真落实党风廉政建设责任制，深入推进党风廉政建设和反腐败斗争。严格执行中央八项规定实施细则和自治区党委、市委的配套规定，高度警惕形式主义、官僚主义新表现，有效约束三公经费支出，坚决防止四风问题反弹回潮。加强财政资金管理，实现自然资源、公共资金、国有资产和领导干部经济责任审计监督全覆盖，严厉查处涉及脱贫攻坚、民生保障等重点领域违法违纪案件，切实维护广大人民群众的根本利益。开展好不忘初心、牢记使命主题教育，自觉保持人民公仆本色，自觉维护人民根本利益，自觉提升思想道德境界，自觉树立良好家风，切实做到干部清正、政府清廉。

　　各位代表，新时代要有新气象，新时代更要有新作为。站在新的历史起点上，踏上实现赤峰跨越崛起的壮阔征程，我们倍感使命光荣、责任重大。让我们更加紧密地团结在以习近平同志为核心的党中央周围，认真学习贯彻落实党的十九大精神，以习近平新时代中国特色社会主义思想为统领，在上级党委和市委的坚强领导下，不忘初心、牢记使命，戮力同心、开拓进取，为决胜全面建成小康社会、开启全面建设现代化赤峰新征程而不懈奋斗！
